# Supplementary material for: Comparison of Wuqinxi Qigong with Stretching on Single- and Dual-Task Gait, Motor Symptoms and Quality of Life in Parkinson’s Disease: A Preliminary Randomized Control Study
Source: Int J Environ Res Public Health. 2022 Jun 30;19(13):8042. doi: 10.3390/ijerph19138042 (PMC9265753; doi:10.3390/ijerph19138042)
Supplement: Supplementary file 1 [file ijerph-19-08042-s001.zip › ijerph-1671744-supplementary.pdf]

CONSORT 2010 checklist of information to include when reporting a randomised trial*

Section/Topic	Item No	Checklist item	Reported on page No
**Title and abstract**			
	1a	Identification as a randomised trial in the title	P1
	1b	Structured summary of trial design, methods, results, and conclusions (for specific guidance see CONSORT for abstracts)	P1
**Introduction**			
Background and objectives	2a	Scientific background and explanation of rationale	P1-2
	2b	Specific objectives or hypotheses	P2
**Methods**			
Trial design	3a	Description of trial design (such as parallel, factorial) including allocation ratio	P2
	3b	Important changes to methods after trial commencement (such as eligibility criteria), with reasons	P2-3
Participants	4a	Eligibility criteria for participants	P2-3
	4b	Settings and locations where the data were collected	P3
Interventions	5	The interventions for each group with sufficient details to allow replication, including how and when they were actually administered	P3-4
Outcomes	6a	Completely defined pre-specified primary and secondary outcome measures, including how and when they were assessed	P4-5
	6b	Any changes to trial outcomes after the trial commenced, with reasons	
Sample size	7a	How sample size was determined	P3
	7b	When applicable, explanation of any interim analyses and stopping guidelines	
Randomisation:			
Sequence generation	8a	Method used to generate the random allocation sequence	P3
	8b	Type of randomisation; details of any restriction (such as blocking and block size)	P3
Allocation concealment mechanism	9	Mechanism used to implement the random allocation sequence (such as sequentially numbered containers), describing any steps taken to conceal the sequence until interventions were assigned	
Implementation	10	Who generated the random allocation sequence, who enrolled participants, and who assigned participants to interventions	P3
Blinding	11a	If done, who was blinded after assignment to interventions (for example, participants, care providers, those	P3

		assessing outcomes) and how	
	11b	If relevant, description of the similarity of interventions	
Statistical methods	12a	Statistical methods used to compare groups for primary and secondary outcomes	P5
	12b	Methods for additional analyses, such as subgroup analyses and adjusted analyses	
**Results**			
Participant flow (a diagram is strongly recommended)	13a	For each group, the numbers of participants who were randomly assigned, received intended treatment, and were analysed for the primary outcome	P5-6
	13b	For each group, losses and exclusions after randomisation, together with reasons	P5
Recruitment	14a	Dates defining the periods of recruitment and follow-up	
	14b	Why the trial ended or was stopped	
Baseline data	15	A table showing baseline demographic and clinical characteristics for each group	P6
Numbers analysed	16	For each group, number of participants (denominator) included in each analysis and whether the analysis was by original assigned groups	P5
Outcomes and estimation	17a	For each primary and secondary outcome, results for each group, and the estimated effect size and its precision (such as 95% confidence interval)	P6-8
	17b	For binary outcomes, presentation of both absolute and relative effect sizes is recommended	
Ancillary analyses	18	Results of any other analyses performed, including subgroup analyses and adjusted analyses, distinguishing pre-specified from exploratory	
Harms	19	All important harms or unintended effects in each group (for specific guidance see CONSORT for harms)	P5
**Discussion**			
Limitations	20	Trial limitations, addressing sources of potential bias, imprecision, and, if relevant, multiplicity of analyses	P9
Generalisability	21	Generalisability (external validity, applicability) of the trial findings	P8-9
Interpretation	22	Interpretation consistent with results, balancing benefits and harms, and considering other relevant evidence	P8-9
**Other information**			
Registration	23	Registration number and name of trial registry	P3
Protocol	24	Where the full trial protocol can be accessed, if available	
Funding	25	Sources of funding and other support (such as supply of drugs), role of funders	P10

*We strongly recommend reading this statement in conjunction with the CONSORT 2010 Explanation and Elaboration for important clarifications on all the items. If relevant, we also recommend reading CONSORT extensions for cluster randomised trials, non-inferiority and equivalence trials, non-pharmacological treatments, herbal interventions, and pragmatic trials. Additional extensions are forthcoming: for those and for up to date references relevant to this checklist, see www.consort-statement.org.

Wuqinxi Qigong exercise [1]

Form 1: Raising the tiger's paws	Move and straighten two arms forward; spread fingers and thumbs and bend them to form "tiger's paws" with palms downwards, eyes on the hands. Turns arm outward, flex fingers and thumbs one by one from little finger to thumb to make fists, and then raise both fists alone the front sides of body to the level of shoulder, thumb to thumbs. Eyes follow the fists.
Form 2: Seizing the prey	Turn right foot outward about 30 degrees, bend and lift knee and move one step forward with heel on ground forming a left empty stance; meanwhile, raise fists up in an arc line and shift them into "tiger's paws", and then push them forward and downward to the sides of knees with palms down; look front below.
Form 3: Colliding with the antlers	Lean body forward about 30 degrees and shift body weight on the left leg, bend left knee and turn tiptoes about 90 degrees outward with sole on the ground while stretching right leg straight with sole on the ground and tiptoes facing forward; meanwhile, change hollow fists into "deer's antler" and move them upward, leftward, and backward in an arc line to the head level with right upper arm touching the ear and palm facing outward while left elbow taken in and touching left side of waist slightly with fingertips facing backward; head turn left and eyes on the right heel.
Form 4: Running like a deer	Lift knee with tiptoes upward, and then take one step forward and make a left bow stance while starching right leg straight; meanwhile, lift hollow fists upward and forward in an arc line alone flanks to the shoulder level with shoulder-width apart, both arms bent slightly, wrists relaxed and elbows sunk with fists downward; look straight ahead.
Form 5: Swaying like a bear	Turn body left and shift body weight backward by flexing right knee and straightening left knee naturally; sway body with arms forward and backward in an arc line, waist leading shoulders and arms forward and backward in an arc line; look forward with the swaying body.
Form 6: Rotating the waist like a bear	Take waist as an axis to rotate upper clockwise while body below hips keeping motionless, and make two clockwise circles continuously meanwhile, take navel as the center and move "bear's paws" in a circle along under, right above, up, left below and down for two circles; eyes follow the ground with rotating of upper body.
Form 7: Lifting the monkey's paws	Put two hands forward, extend ten fingers and separate them from one another, and then bend wrists and collect fingers together forming "monkey's hooked paws". Lift two hands to the chest level, shrug shoulders, clamp arms, erect waist, contract abdomen, and clench anus; meanwhile, lift up heels and turn head to the left, and look directly to the left.
Form 8: Picking fruit	Move right foot right forward about 45 degrees and shift body weight to the right leg, straighten right knee while taking left leg in and straightening left knee with tiptoes touching the ground; meanwhile, move right palm up in an arc line to the right ear with keeping a little higher than shoulders; straighten left arm and turn "monkey's hooked paw" into a palm, and move it from backward to upward and forward with wrist erected and fingertips together as if a monkey is picking fruits from a tree; eyes on the left wrist.
Form 9: Stretching upward	Take a semi-squatting position with two palms overlapping forward, left hand on top. Straighten two elbows naturally and raise two hands above head with upper arms touching ears, wrists erected and fingers pressed and forward; throw out chest, contract abdomen and neck, lift shoulders, and lean upper body forward slightly; look front below.
Form 10: Flying like a bird	Take a semi-squatting position, put two hands in front of abdomen with palms slanting and fingertips downward, and facing each other; look front below. Straighten right knee and stand on it, lift left knee with tiptoes pointing downward; meanwhile, raise two palms to a-little-higher-than-shoulder level with palms facing downward like a bird flying, hands in the form of "bird's wing"; look straight ahead.

Stretching exercise [2]

**Upper limb muscles**	
Deltoid	Stretch the front bundle of the deltoid muscle for 10-20 seconds with your hands straight behind your body and fingers interlocked.
Biceps	Practitioner Uses horizontal surface, such as a railing, a dancing pole, or the back of a chair, or can practice with a door handle closed. The practitioner stands (or kneels on one leg) with the upper arm straight and palm inward, extending the upper arm as far back as possible, keeping the body upright. Relax the extended upper arm on the level or grab the door handle.
Triceps	Stand vertically with back and neck straight, or take a sitting position. Bend your shoulder and elbow and try to touch your opposite scapular. The upper arm should be as close to the ear as possible, with the back of the humerus pointing forward rather than turning outwards. This will maximize the length of the triceps.
**Low limb muscles**	
Iliopsoas	Step with one foot and the other foot back, keeping your pelvis forward, and move your body and pelvis forward as you stretch.
Quadriceps	Sit on the mat with your right leg bent and your right heel outside your right hip. Then bend your left foot and bring the heart of your left foot to the inside of your right thigh. Stretch your feet back, keep your feet straight and your ankles bent. Slowly lean back until you can feel an easy stretch, supporting your body with your hands and maintaining balance. Stretch the left thigh in the same way.
Hamstring,	Stand with one leg straight in front of you, feet on the ground, toes hooked up above and in front of your knees, hands on your thighs, elbows slightly bent for balance. Bend the other leg down (not down) until the hamstring feels stretched in the back of the thigh. Hold for 10 to 20 seconds and relax. Repeat the stretch 5 times on each leg.
Tibialis anterior	Sit on a chair or bench and lower your knees, then place your toes on the ground behind you, leaning forward slightly and pressing them into the ground. Hold for a maximum of 20 seconds and repeat on the other leg.
Gastrocnemius	Stand with legs spread and step forward and back, pressing your hips down. Place your hands parallel to your shoulders in a bow position. Hold for 10 seconds until pain appears. Switch sides and repeat 5 times on each side. When pressing your legs, make sure your back legs are as straight as possible.
**Trunk muscles**	
Pectoralis major	Sit in a chair with your hands crossed behind your head and the top of the chair at mid-chest height. Breath in, lean back, pull your arms back.
Erector spinae	Lie on your back on the mat in a straight line with your hands and arms outstretched at shoulder height. Breathe in, lift your feet 90 degrees, bend your knees together, bring your thighs to your abdomen, and wrap your arms around your calves. Breath out, drop your legs to the floor to the left, turn your head to the right, rest your shoulders on the mat as far as possible. Breathe in, bring your knees and head back into alignment, and do the reverse.

[1]Li Ying. Health Qigong Wuqinxi. 1st ed.; Dalian Maritime University Press.; Dalian, China, 2016, 45-95.

[2]Bob Anderson. Stretching: 40th Anniversary Edition. Shelter Publication Inc., U.S, 2020,
